# A novel BMP-2–loaded hydroxyapatite/beta-tricalcium phosphate microsphere/hydrogel composite for bone regeneration

**DOI:** 10.1038/s41598-021-96484-4

**Published:** 2021-08-19

**Authors:** Daisuke Tateiwa, Shinichi Nakagawa, Hiroyuki Tsukazaki, Rintaro Okada, Joe Kodama, Junichi Kushioka, Zeynep Bal, Yuichiro Ukon, Hiromasa Hirai, Takashi Kaito

**Affiliations:** 1 Department of Orthopaedic Surgery, Osaka University Graduate School of Medicine, 2-2 Yamadaoka, Suita, Osaka 565-0871 Japan; 2 Department of Orthopaedic Surgery, Mino Municipal Hospital, Mino, Osaka Japan; 3 Department of Orthopaedic Surgery, Kansai Rosai Hospital, Amagasaki, Hyogo Japan

**Keywords:** Bone, Drug delivery

## Abstract

Although bone morphogenetic protein (BMP) has potent osteoinductivity, the potential adverse events attributed to its burst release prevent its widespread clinical application. Therefore, there is a strong need for BMP delivery systems that maximize osteoinductivity while preventing adverse effects. We evaluated the bone-regenerating potential of NOVOSIS putty (NP), a novel composite combining hydroxyapatite, beta-tricalcium phosphate microsphere/poloxamer 407-based hydrogel, and recombinant human (rh) BMP-2. In vitro assessment of release kinetics by enzyme-linked immunosorbent assay demonstrated sustained release of rhBMP-2 from NP and burst release from collagen sponge (CS), and in vivo assessment of release kinetics by longitudinal tracking of fluorescently labeled rhBMP-2 showed a longer biological half-life of rhBMP-2 with NP than with CS. Furthermore, osteogenic gene expression in MC3T3-E1 cells was significantly higher after co-culture with NP than after co-culture with CS, suggesting that the sustained release of rhBMP-2 from NP effectively contributed to the differentiation of osteoblasts. In a rat spinal fusion model, the volume and quality of newly formed bone was higher in the NP group than in the CS group. Use of NP results in efficient bone regeneration through sustained release of rhBMP-2 and improves the quality of BMP-induced bone.

## Introduction

Bone morphogenetic proteins (BMPs) play important roles in osteogenesis and bone metabolism^1–3^. Among the BMP subtypes, BMP-2 has the most potent osteoinductive capacity, and the US Food and Drug Administration has approved the use of recombinant human BMP-2 (rhBMP-2) with absorbable collagen sponges (CSs) for anterior lumbar interbody fusion^4^. Clinical trials of absorbable CSs with milligram-order supraphysiological doses of rhBMP-2 for anterior lumbar interbody fusion showed better bone fusion rates than those achieved with standard iliac crest bone grafting^5,6^. However, high-dose rhBMP-2 is associated with adverse effects, including inflammation, soft tissue edema, seroma, and unintended ectopic bone formation, which prevent its widespread clinical application^7,8^.

To mitigate these adverse effects, carriers have been developed that provide sustained release of BMP-2; in addition, these carriers have other desirable properties, including easy handling^7–10^, good mechanical strength^11–13^, and spatial control of new bone formation^11,14^. The previously studied BMP carriers can be categorized into natural polymers (e.g., collagen, alginate, and gelatin), synthetic polymers (e.g., polyglycolic acid, poly-lactic-co-glycolic acid, and polylactic acid-polyethylene glycol), ceramics (e.g., hydroxyapatite [HA], beta-tricalcium phosphate [β-TCP], and biphasic calcium phosphate), and ceramic/polymer composites^7,15–17^. Recently, ceramic/polymer composites, which have the benefits of both materials, have attracted particular attention as BMP delivery systems^7,16^.

We developed a novel ceramic/polymer composite (NOVOSIS putty [NP]) by combining HA granules, a β-TCP microsphere/poloxamer 407-based hydrogel (β-TCP/hydrogel), and rhBMP-2. The HA granules provide mechanical strength, and the β-TCP/hydrogel ensures the sustained release of rhBMP-2 and also provides excellent handling^18–20^. Furthermore, we expected that using two types of ceramics with different levels of biodegradability would have an additive effect on osteogenesis^21,22^. During the bone formation period, the low-biodegradable HA granules can maintain scaffold volume by preventing soft tissue invasion^21,23^, whereas the β-TCP/hydrogel is gradually resorbed and efficiently replaced by new bone^20,21^.

In the present study, we investigated the in vitro and in vivo release kinetics of rhBMP-2 from NP and CS and the effects of rhBMP-2 on MC3T3-E1 cells (pre-osteoblasts). Furthermore, we used the rat spinal fusion model to compare the in vivo efficacy of NP and CS as BMP delivery systems.

## Results

### In vitro release kinetics of rhBMP-2

**Figure 1:**
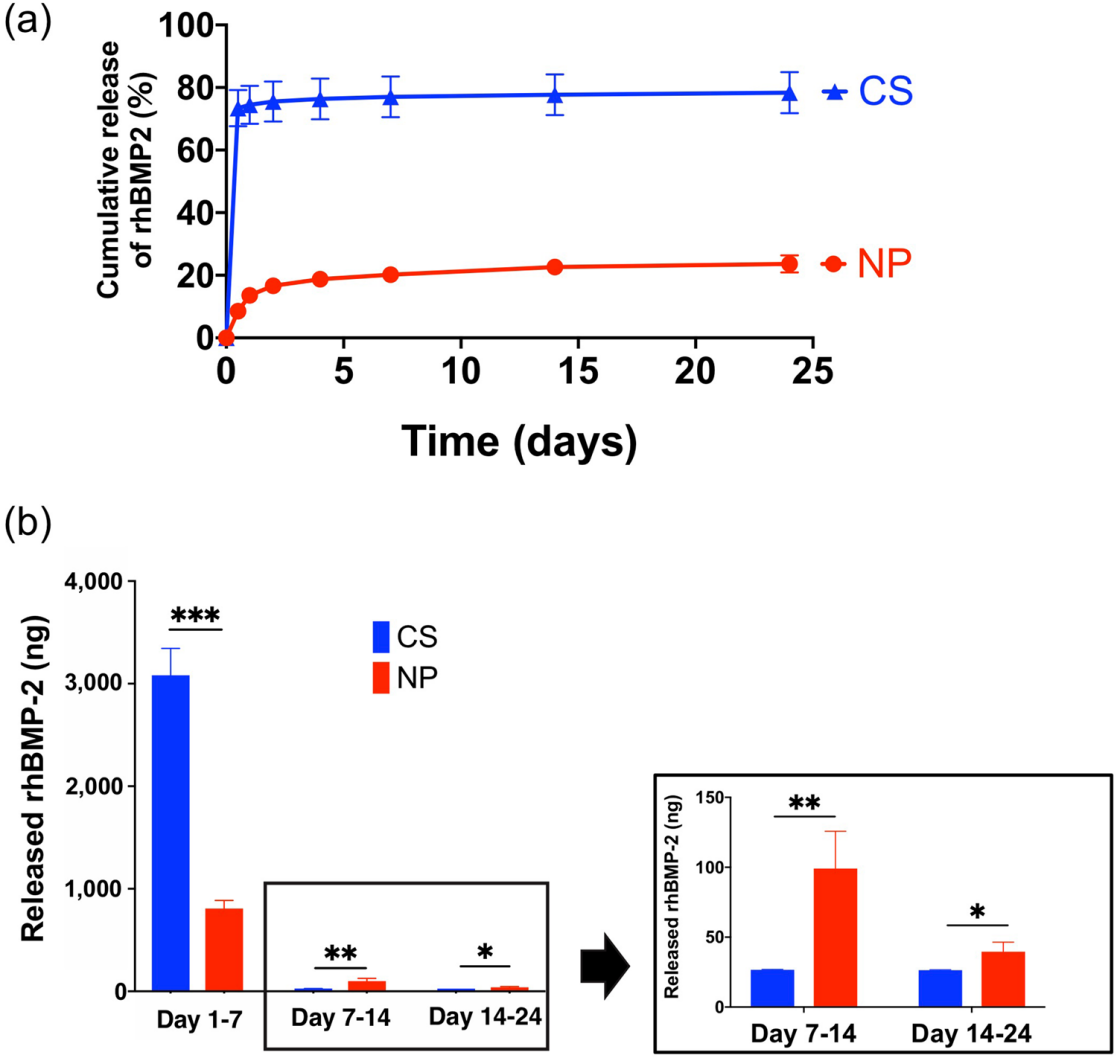
In vitro release kinetics of recombinant human bone morphogenetic protein 2 (rhBMP-2). (**a**) Release kinetics of rhBMP-2 from NOVOSIS putty (NP) and collagen sponge (CS) for up to 24 days (12 h and 1, 2, 4, 7, 14, and 24 days). NP showed less initial release of rhBMP-2 and sustained release. (**b**) From days 1 to 7, CS released significantly more rhBMP-2 than NP did (CS = 3082 ng, NP = 807.3 ng; data represent mean ± S.D., each *n* = 3; ***p = 0.0001 by Student’s *t* test), but NP released significantly more on days 7–14 (CS = 26.7 ng, NP = 99.1 ng; data represent mean ± S.D., each *n* = 3; **p = 0.0093 by Student’s *t* test) and days 14–24 (CS = 26.4 ng, NP = 39.6 ng; data represent mean ± S.D., each *n* = 3; *p = 0.0299 by Student’s *t* test).

The amounts of rhBMP-2 released from CS and NP were evaluated by enzyme-linked immunosorbent assay (ELISA) (Fig. 1a). Until day 24, the total amounts of rhBMP-2 released from CS and NP were 3.14 μg and 0.90 μg, respectively, which were 78.4% and 22.5%, respectively, of the initially loaded dose (4 μg). From days 1 to 7, significantly more rhBMP-2 was released from CS than from NP; CS released most of the rhBMP-2 on day 1, whereas NP released it more gradually. The amounts of rhBMP-2 released from days 7 to 14 and from days 14 to 24 were significantly higher from NP than from CS (Fig. 1b).

### In vivo release kinetics of fluorescently labeled rhBMP-2

**Figure 2:**
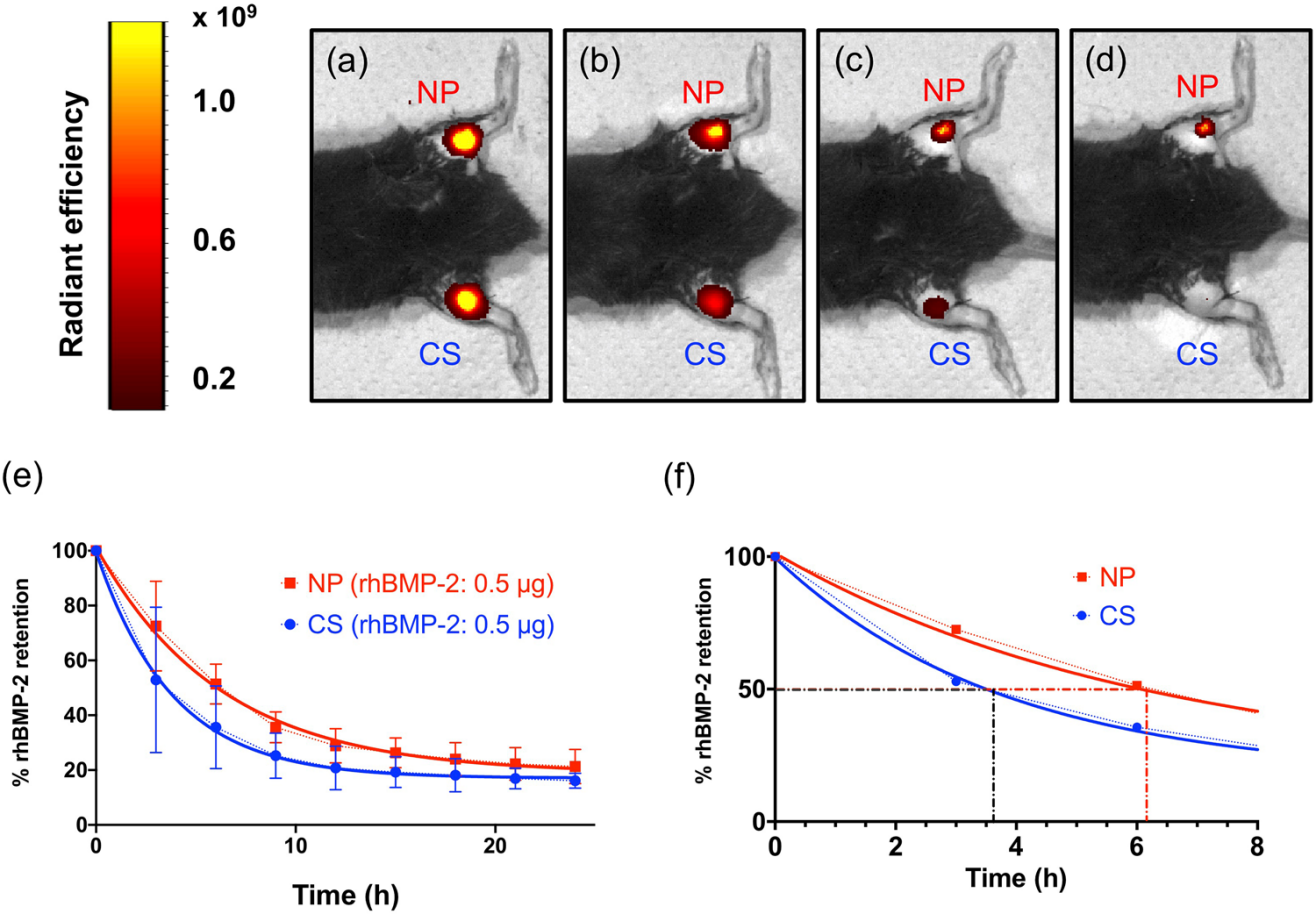
In vivo release kinetics of fluorescently labeled recombinant human bone morphogenetic protein 2 (rhBMP-2)*. *In vivo imaging of fluorescently labeled rhBMP-2 released from collagen sponge (CS) and NOVOSIS putty (NP) (**a**–**d**): (**a**) 0 h, (**b**) 6 h, (**c**) 12 h, and (**d**) 18 h. The images were created by IVIS Living Image Software (version 4.2, Caliper Life Sciences, Inc.) (**e**) In vivo release kinetics based on fluorescence quantification at the implantation sites. (**j**) Biological half-life of rhBMP-2 (CS = 3.8 h, NP = 6.2 h).

In vivo imaging of fluorescently labeled rhBMP-2 depicted the in vivo release kinetics of rhBMP-2 (Fig. 2a–d). The fluorescent signal at each measurement was normalized to the initial measurement, and an exponential decay curve was created (Fig. 2e). The biological half-life of rhBMP-2 was 3.8 h in CS and 6.2 h in NP (Fig. 2f), suggesting that NP enables better sustained release of rhBMP-2 in vivo than CS.

### In vitro co-culture experiments

#### Cytotoxicity evaluation with cell counting kit-8 (CCK-8)

**Figure 3:**
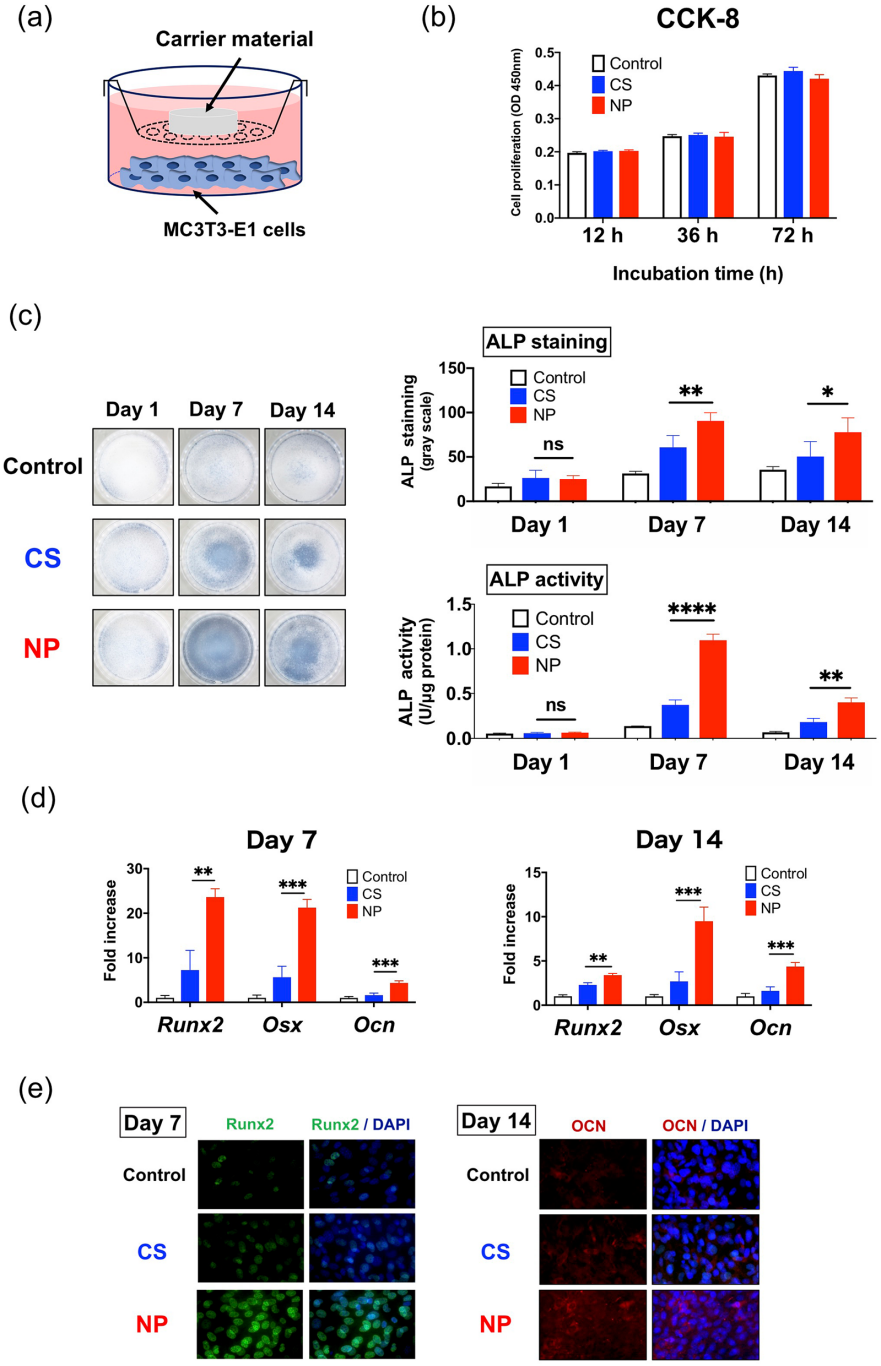
In vitro co-culture experiments. (**a**) Schematic presentation of the chamber co-culture of MC3T3-E1 cells and carrier material. (**b**) Cytotoxicity testing of carrier materials by Cell Counting Kit-8 found no apparent cytotoxicity of collagen sponge (CS) or NOVOSIS putty (NP) (data represent mean ± S.D., each *n* = 3; no significant differences in one-way analysis of variance [ANOVA] followed by Bonferroni multiple comparison test). (**c**) Osteogenic differentiation of MC3T3-E1 cells with recombinant human bone morphogenetic protein 2 (rhBMP-2)–loaded CS and NP. Representative alkaline phosphatase (ALP) staining images (days 1, 7, and 14; left-hand panel) and quantification of ALP staining and activity (data represent mean ± S.D., each *n* = 3; *p < 0.05, **p < 0.01, ****p < 0.0001, and ns, not significant by one-way ANOVA followed by Bonferroni multiple comparison test; right-hand panel). (**d**) Expression levels of the osteogenic genes Runx2, Osx, and Ocn on days 7 and 14 (data represent mean ± S.D., each *n* = 3; **p < 0.01 and ***p < 0.001 by one-way ANOVA followed by Bonferroni multiple comparison test). (**e**) In the immunocytochemical analysis, the protein expressions of Runx2 (green; left-hand panels) and Ocn (red; right-hand panels) were more enhanced in the MC3T3-E1 cells co-cultured with NP than in those co-cultured with CS (images are shown for Runx2 on day 7 and Ocn on day 14). DAPI, 4′,6-diamidino-2-phenylindole.

The cytotoxicity of NP and CS was evaluated by investigating the proliferation of MC3T3-E1 cells under co-culture with NP or CS not containing rhBMP-2 (Fig. 3a). After 12, 36, and 72 h of co-culture, we found no statistically significant difference in cell proliferation between the control (chamber only), CS, and NP (Fig. 3b).

#### Alkaline phosphatase (ALP) staining and activity

Co-culture of MC3T3-E1 cells with CS or NP containing 1 μg of rhBMP-2 to evaluate ALP staining and activity showed no significant difference on day 1, but both staining and activity were significantly higher with NP than with CS on days 7 and 14 (Fig. 3c). The co-culture was performed for 14 days because most of the rhBMP-2 was released from the composites within 14 days in ELISA experiments.

#### Real-time polymerase chain reaction (PCR) assay of osteogenic genes

The expression levels of runt-related transcription factor 2 (Runx2), osterix (Osx), and osteocalcin (Ocn) were significantly higher with NP than with CS on both days 7 and 14 (Fig. 3d), suggesting that the sustained release of rhBMP-2 from NP effectively contributes to the differentiation of osteogenic cells.

#### Immunocytochemical analysis

Protein expression analysis by immunocytochemical analysis confirmed the superior effects of NP on differentiation of osteogenic cells in MC3T3-E1 cells: After 7 days of co-culture, expression of Runx2 (green), an early differentiation marker of osteoblasts, was considerably more enhanced with NP than with CS (Fig. 3e); after 14 days of co-culture, expression of Ocn (red), a late differentiation marker, was also considerably more enhanced with NP than with CS.

### Posterolateral spinal fusion model

#### High-resolution micro-computed tomography (micro-CT) analysis

**Figure 4:**
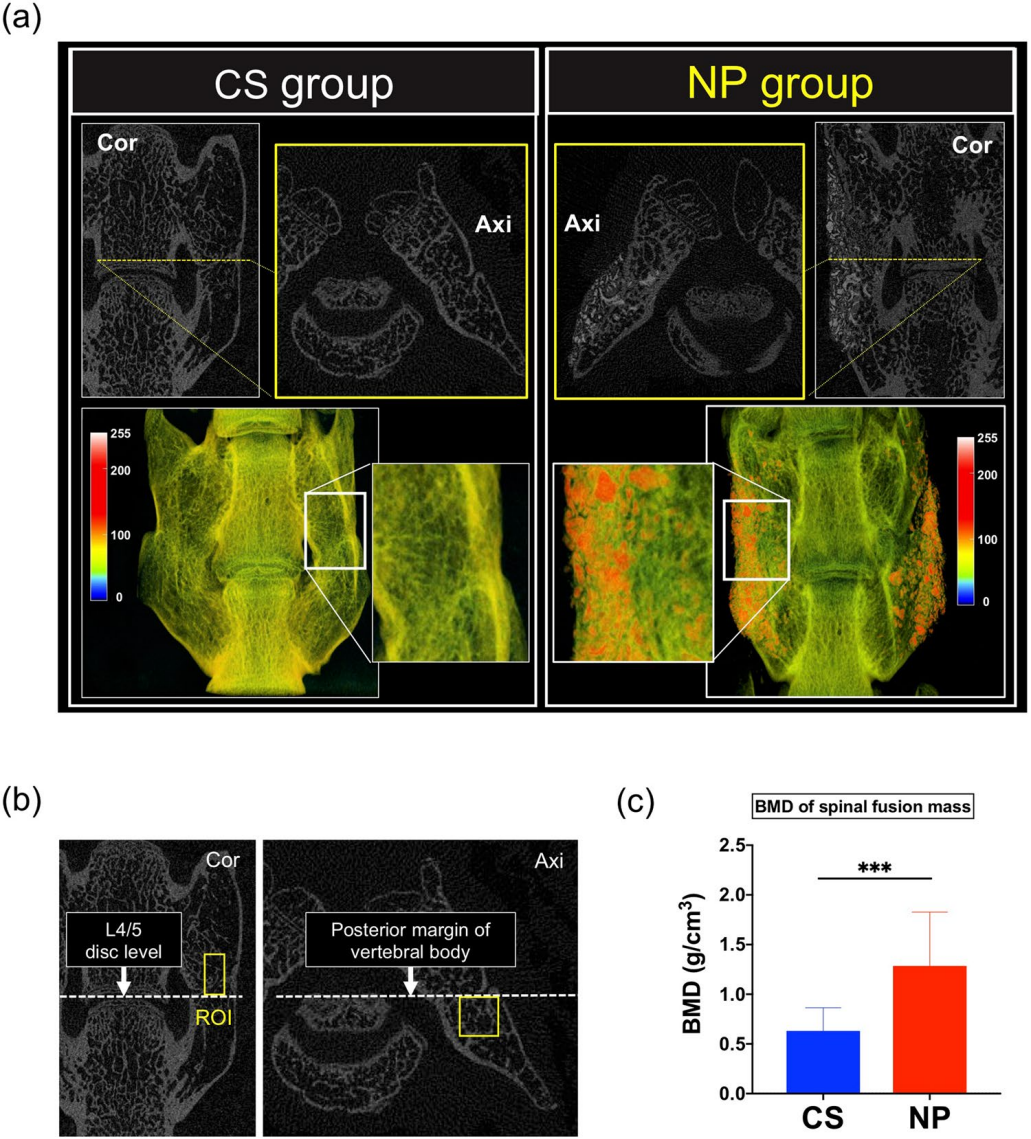
High-resolution micro-computed tomography (micro-CT) analysis. (**a**) Representative images of high-resolution micro-CT analysis of the collagen sponge (CS) and NOVOSIS putty (NP) groups. Grayscale images were transformed into pseudo-color images by CT-Analyser (CTAn) software (version 1.17, https://www.bruker-microct.com). Cor, coronal view; Axi, axial view. (**b**) Region of interest (ROI) for bone mineral density (BMD) measurement. A 1 × 1 × 2-mm^3^ ROI passing through the center of the spinal fusion mass was placed cranial to the L4/L5 disc in the coronal plane and ventral to the posterior margin of the vertebral body in the axial plane. (**c**) The BMD of the new bone was significantly higher in the NP group than in the CS group (0.63 g/cm^3^ vs. 1.29 g/cm^3^, respectively; data represent mean ± S.D.; ***p = 0.0001 by Student’s *t* test).

The final fusion rate at postoperative week 8 was 72.7% (*n* = 8/11) in the CS group and 81.8% (*n* = 9/11) in the NP group (*p* > 0.999 by Fisher’s exact test). Micro-CT images of the fused spine segments in the NP group showed that HA was incorporated into the new bone to form a spinal fusion mass between the L4 and L5 transverse processes (Fig. 4a). The bone mineral density (BMD) of the fusion mass was significantly higher in the NP group than in the CS group (0.63 g/cm^3^ vs. 1.29 g/cm^3^, respectively; ***p < 0.001; Fig. 4b,c).

#### Manual palpation test

Manually assessed spinal fusion rates did not statistically differ between the CS (*n* = 8/11, 72.7%) and NP (*n* = 9/11, 81.8%) groups (*p* > 0.999, difference was not significant by Fisher’s exact test).

#### Histological analysis

**Figure 5:**
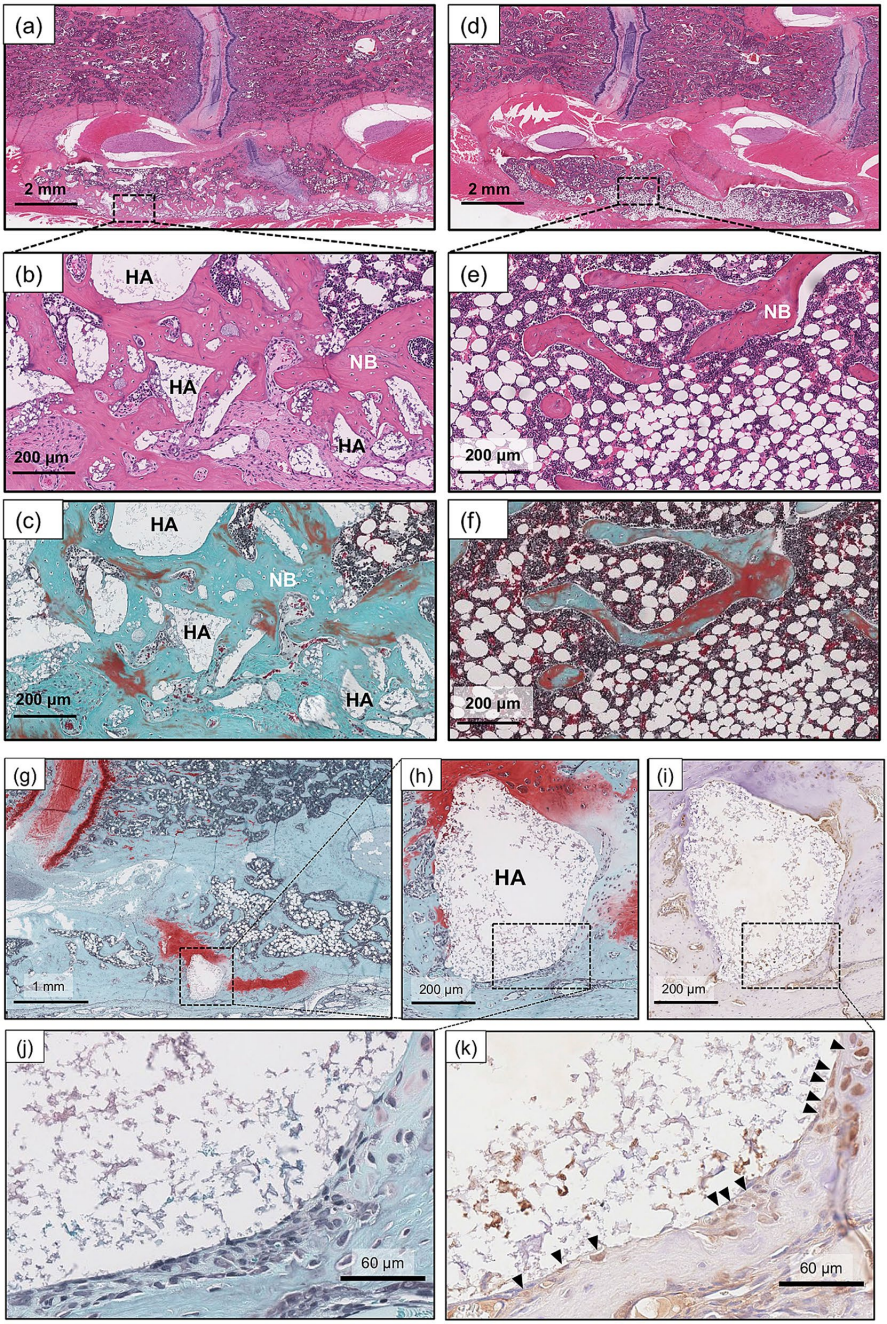
Histological analysis of the NOVOSIS putty (NP) and collagen sponge (CS) groups. (**a**–**c**) In the NP group, hydroxyapatite (HA) and new bone intermingled to form the spinal fusion mass. Some of the beta-tricalcium phosphate (β-TCP) microspheres were resorbed. (**d**–**f**) In the CS group, fatty marrow was predominant inside the fusion mass. (**g**–**k**) HA served as a scaffold for cell adhesion. HA surfaces were covered with osteocalcin-positive osteoblast-like cells (k, black arrows). (**a**,**b**,**d**,**e**) H&E staining; (**c**,**f**) Goldner’s Masson trichrome staining; (**g**–**i**) safranin-O staining; and (**i**,**k**) immunohistochemistry for osteocalcin. *NB* new bone, *HA* hydroxyapatite.

In the NP group, the fusion mass was filled with abundant new bone. Some of the β-TCP microspheres were resorbed and replaced by new bone, and unabsorbed HA was incorporated into the new bone (Fig. 5a–c). The finding that HA surfaces were covered with osteocalcin-positive, osteoblast-like cells suggests that the unabsorbed HA served as a scaffold for cell adhesion (Fig. 5g–k). In contrast, newly formed bone was scarce in the CS group, and fatty marrow occupied a large amount of space inside the fusion mass (Fig. 5d–f).

#### Quantification of the newly formed bone area in the fused spinal fusion masses

**Figure 6:**
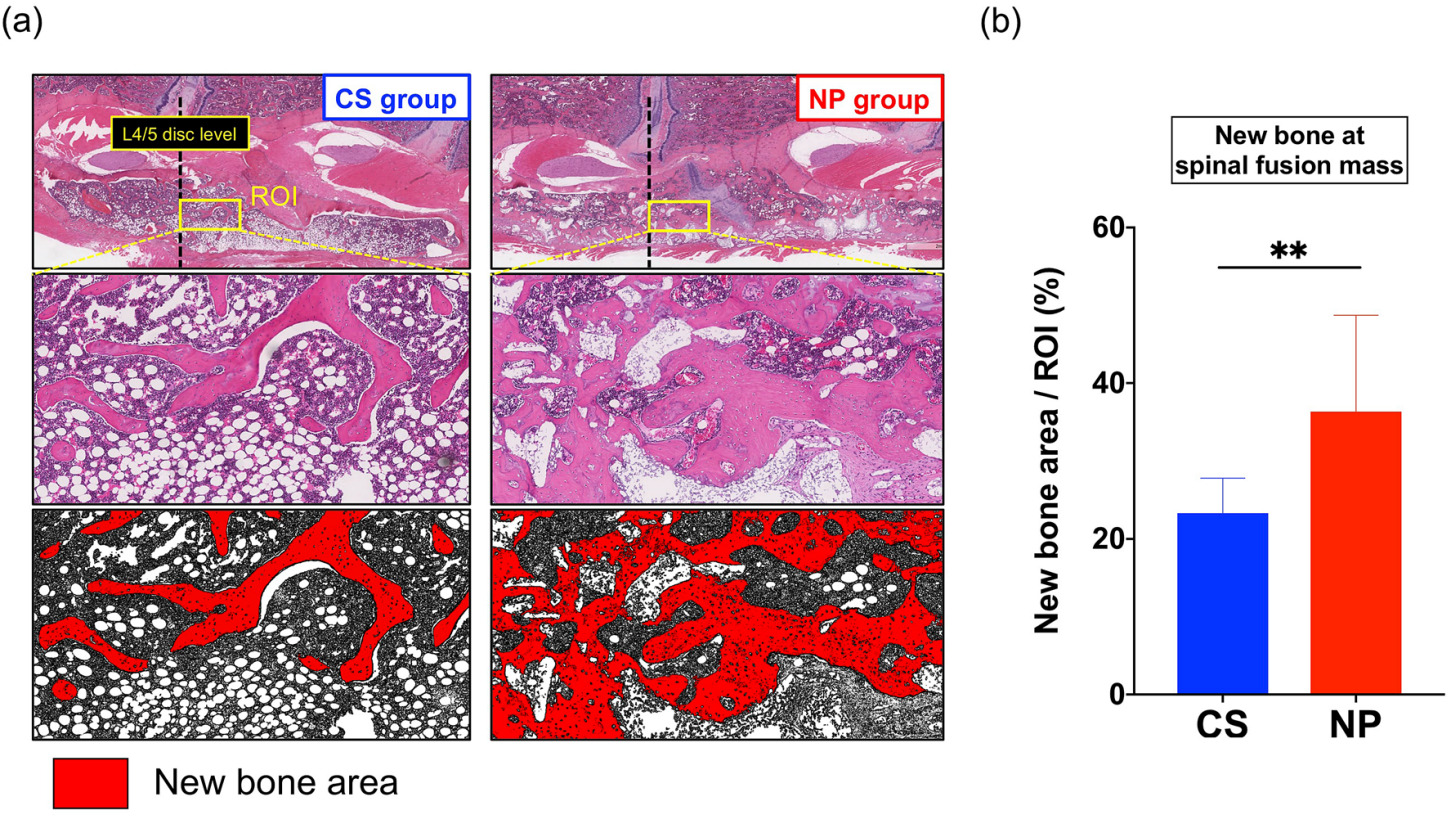
Quantification of the new bone area in the fusion mass. (**a**) Comparison of new bone area inside the spinal fusion mass in histological sections. A 1 × 2-mm^2^ region of interest (ROI; cranial to the L4/L5 disc) was extracted from the newly formed fusion mass. The new bone area (red) was color coded with ImageJ software (version 1.52q, U. S. National Institutes of Health; https://imagej.nih.gov/ij/). (**b**) The percentage of new bone area in the ROI was significantly higher in the NOVOSIS putty (NP) group than in the collagen sponge (CS) group (23% vs. 36%; data represent mean ± S.D.; **p < 0.0086 by Student’s *t* test).

The percentage of the new bone area in the region of interest (ROI) was significantly higher in the NP group (*n* = 9; 36%) than in the CS group (*n* = 8; 23%; **p < 0.01; Fig. 6a,b).

## Discussion

This study demonstrated the sustained release of rhBMP-2 from NP both in vitro and in vivo. When implanted into a rat spinal fusion model, NP demonstrated better bone regeneration capacity than CS and higher bone volume and quality. The superior bone regeneration by NP was considered to be related to both the sustained release of rhBMP-2 and the higher quality of BMP-induced bone.

In this study, in vitro release kinetics demonstrated that NP attenuates the initial burst release of rhBMP-2 and provides sustained release of rhBMP-2. This release kinetics is similar to that of another ß-TCP microsphere/hydrogel composite^18^. Co-culture experiments showed that ALP activity and osteogenic gene expression levels were significantly higher with NP. The rapid release of rhBMP-2 from CS may be of insufficient duration to enhance osteogenic activity of co-cultured cells^24,25^, and the sustained release of rhBMP-2 from NP likely contributes more effectively to osteogenic cell differentiation. The combination of ceramic and polymer carriers in NP is considered to contribute to its release characteristics. HA is a low-biodegradable material that has high affinity to rhBMP-2, allowing it to serve as a long-term carrier^26,27^. However, the high affinity of HA to rhBMP-2 and the low biodegradability of HA mean that a single application releases only limited amounts of rhBMP-2 and insufficiently induces bone formation^26,27^. To overcome this problem, we combined HA with β-TCP microsphere/hydrogel, which has been shown to release rhBMP-2 in a sustained manner without an initial burst^18–20^. Poloxamer 407, which shows thermo-reversible gelation, forms a gel state at body temperature and promotes slow and sustained drug release^28–30^, resulting in excellent bone regeneration as a BMP carrier^31^. The β-TCP microspheres will also release rhBMP-2 slowly^18,20^. The biodegradable β-TCP/hydrogel can continuously release rhBMP-2; however, it may not be able to retain rhBMP-2 for long periods, depending on the rate of degradation. The combination of HA and β-TCP/hydrogel can compensate for the shortcomings of each material by utilizing the strengths of each, resulting in the sustained release of rhBMP-2.

**Figure 7:**
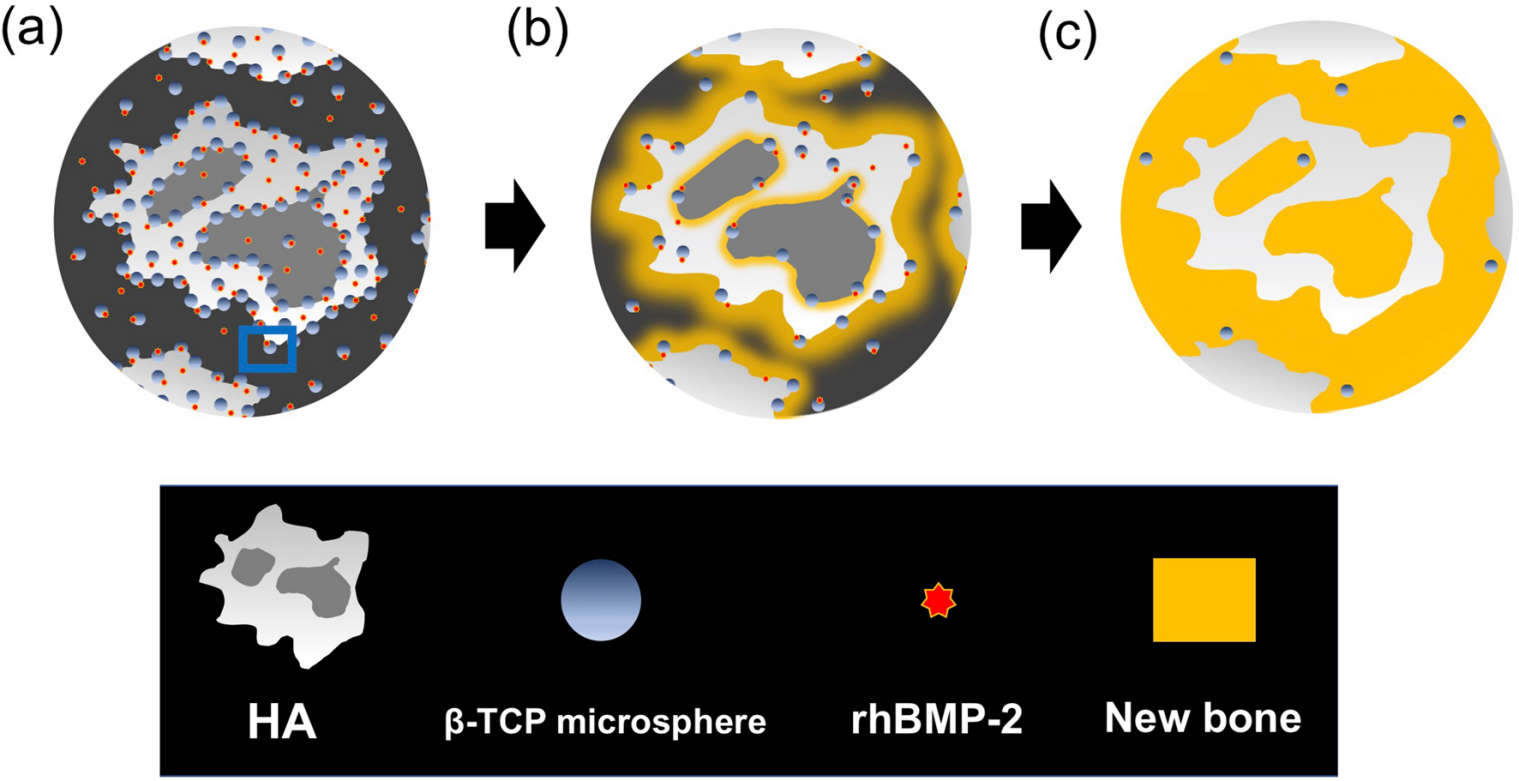
Schematic showing the osteogenic process of NOVOSIS putty (NP) (**a**) Composite after implantation. (**b**) Recombinant human bone morphogenetic protein 2 (rhBMP-2) is slowly released, and some of the beta-tricalcium phosphate (β-TCP) microspheres are absorbed and replaced by new bone. (**c**) The unabsorbed hydroxyapatite (HA) and abundant new bone are mixed to form a high-BMD spinal fusion mass.

NP also improves the quality of BMP-induced bone compared with CS. The amount of new bone at the spinal fusion mass was significantly larger in the NP group than in the CS group, where trabecular bone was scarce and abundant fatty marrow occupied the fusion mass. The burst release of high-dose BMP-2 from CS induces the formation of structurally abnormal bone with scant trabecular bone and abundant fatty bone marrow^32^; however, NP provides gradual release of rhBMP-2 rather than an initial burst release and leads to formation of a spinal fusion mass with more new bone and less fatty marrow compared with CS. Furthermore, histological analysis showed that the low-biodegradable HA secured a space for bone formation and served as a long-term scaffold for cell adhesion. In contrast, some of β-TCP/hydrogel was resorbed and efficiently replaced by new bone, resulting in the formation of a high-BMD spinal fusion mass with a mixture of unabsorbed HA and abundant new bone (Fig. 7a–c).

This study has several limitations. First, spinal fusion in a quadrupedal rodent model is different from that in humans in terms of biomechanics and biological response^33–36^. Hence, care should be taken when extrapolating the results of this study to humans. Second, the study did not investigate the effects of NP on rhBMP-2–related complications. Although the complications are difficult to reproduce in this rat spinal fusion model^33,36^, the sustained release of rhBMP-2 from NP can be expected to attenuate the incidence of complications. Another study is currently underway in a rat coccygeal interbody fusion model to elucidate the effects of NP on the incidence of complications. Third, biomechanical testing of bone fusion was performed by a manual palpation test because of the size and anatomical complexity of the rat spine. Nevertheless, manual palpation is validated as a substitute method for biomechanical testing^37^.

In conclusion, NP showed better bone regeneration capacity than CS through sustained release of rhBMP-2 and higher quality of BMP-induced bone.

## Methods

### Characterization of NP materials

NP was provided by CGBio Co., Ltd (Seoul, Korea).

**Figure 8:**
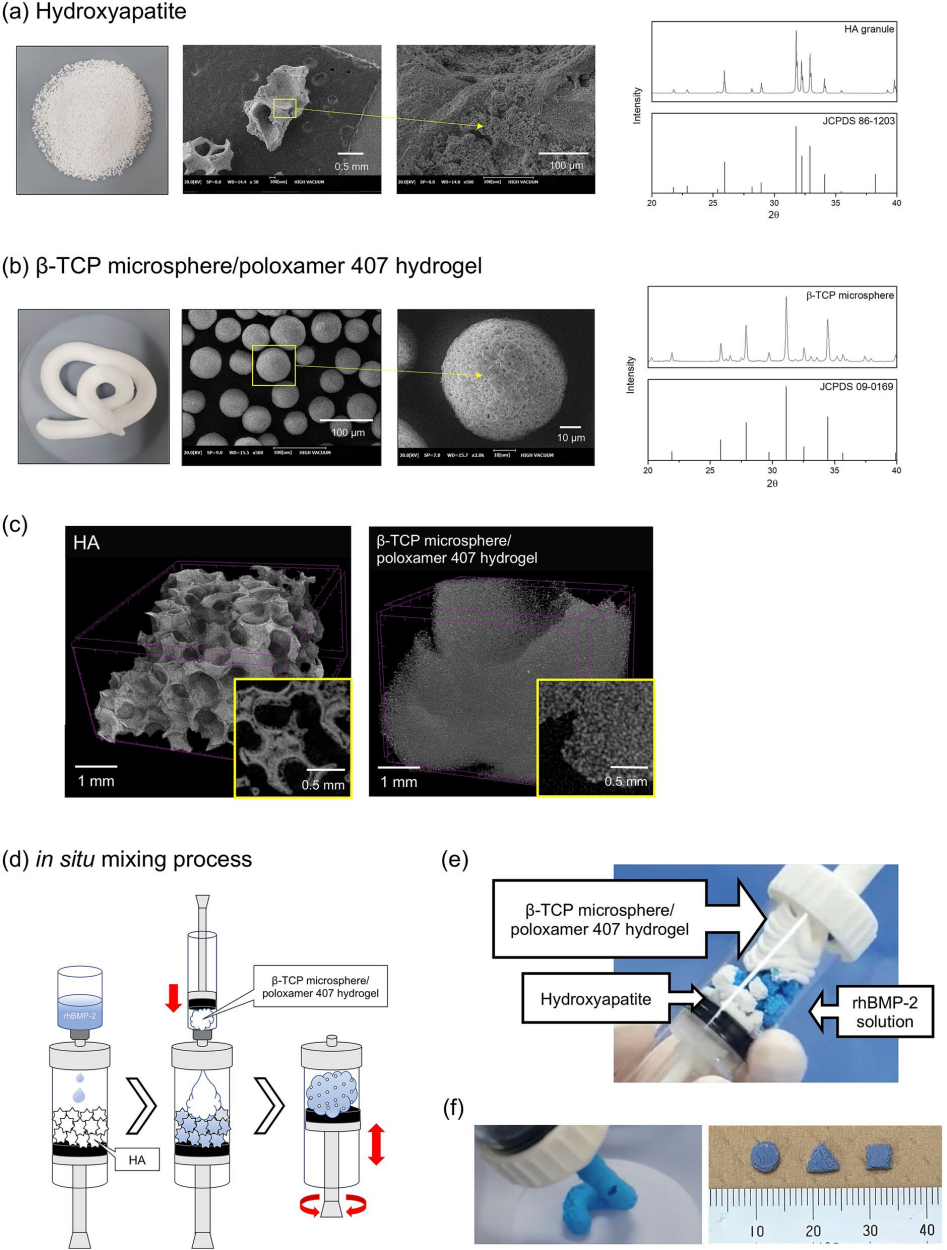
The characterization of NOVOSIS putty materials. (**a**) Hydroxyapatite (HA) granules: scanning electron microscopy (SEM) images (on the left) and X-ray diffraction (XRD) patterns (on the right). (**b**) β-TCP microsphere/poloxamer 407-based hydrogel (β-TCP/hydrogel): SEM images and XRD patterns. (**c**) High-resolution micro-computerized tomography images of HA (left) and β-TCP/hydrogel (right). (**d**) Schematic illustration of the in situ mixing process. (**e**) HA granules after soaking in rhBMP-2 solution (stained blue) and β-TCP/hydrogel are mixed in a syringe. (**f**) Composite is injectable and moldable.

1. HA granules: Each granule was 3.0–6.0 mm in diameter, with approximately 70% porosity and 99% interconnectivity. The X-ray diffraction (XRD) pattern of the granules was consistent with the theoretical XRD pattern of HA specified by the Joint Committee on Powder Diffraction Standards (JCPDS) (Fig. 8a,c).
2. β-TCP microsphere/poloxamer 407-based hydrogel: Microspheres (45–75 μm diameter) were formed into globular shapes by spray drying, and a porous structure (68% porosity) was created by sintering at 1050 °C. The XRD pattern of the β-TCP microsphere was consistent with the theoretical XRD pattern of β-TCP specified by the JCPDS (Fig. 8b,c). Poloxamer 407, a biodegradable, non-toxic polymer, exhibits thermo-reversible gelation, i.e., the aqueous solution is liquid at low temperature and forms a semisolid gel at body temperature^28,29^, so it promotes slow and sustained drug release^28,30^. Poloxamer 407 hydrogel and β-TCP microspheres were mixed at a 1:1 weight ratio.

HA granules (6 g) were soaked in 1.2 mL of rhBMP-2 solution, crushed into small pieces and mixed with 9 g of β-TCP/hydrogel to form a homogeneous, putty-type composite (Fig. 8d,e) that was injectable and moldable and therefore easy to handle during surgery (Fig. 8f).

### In vitro release kinetics of rhBMP-2

Three NP and 3 CS (CollaCote; Zimmer Dental, Carlsbad, CA, USA), each containing 4 μg rhBMP-2, were immersed in 1 mL of phosphate-buffered saline (PBS) and incubated at 37 °C under constant agitation. After centrifugation, the entire supernatant (1 mL) was collected and refilled with the same amount (1 mL) of PBS after 12 h and 1, 2, 4, 7, 14, and 24 days^38^. The amount of released rhBMP-2 was quantified by ELISA^38^. The standard curve was based on the rhBMP-2 used in this study.

### In vivo release kinetics of fluorescently labeled rhBMP-2

rhBMP-2 was fluorescently labeled with an amine-reactive (NHS ester) near-infrared fluorochrome (VivoTag-S 750), as described previously^14^. CS containing 0.5 μg of labeled rhBMP-2 was implanted subcutaneously into the left lower leg of 6-week-old male C57BL/6J mice (*n* = 5), and NP containing 0.5 μg of labeled rhBMP-2 was implanted into the right lower leg of the same mice. Fluorescence imaging was performed with an in vivo imaging system (IVIS) after 0, 3, 6, 9, 12, 15, 18, 21, and 24 h. Total fluorescent count and radiant efficiency were measured, and the fluorescence signal was normalized to values at 0 h^14^. Finally, imaging data were analyzed with IVIS Living Image Software (version 4.0, Caliper Life Sciences, Inc., Waltham, MA, USA). The Animal Experimental Committee of Osaka University Graduate School of Medicine approved all animal studies (approval number: 30-076-003), which were performed in accordance with ARRIVE guidelines and the National Institutes of Health Guide for the Care and Use of Laboratory Animals^39^.

### In vitro co-culture experiments

#### Cell culture and co-culture model

MC3T3-E1 cells were cultured in growth medium containing α-modified Eagle’s medium, 10% FBS, and 1% antibiotic-antimitotic in a humidified atmosphere of 5% CO_2_ at 37 °C. Then, culture insert chambers (Falcon Cell Culture Inserts; pore size = 0.4 μm) containing CS or NP with or without rhBMP-2 were suspended above each well. A vacant chamber (chamber-only) group was used as a control.

#### Cytotoxicity test

The cytotoxicity of CS and NP not containing rhBMP-2 was evaluated with the CCK-8 assay. MC3T3-E1 cells were seeded in 24-well tissue culture plates (6.0 × 10^3^ cells/well). After 12-h incubation to allow for cell attachment, the chambers containing CS or NP without rhBMP-2 were suspended above each well; cell proliferation was assessed after 12, 36, and 72 h of co-culture.

#### Osteogenic differentiation

MC3T3-E1 cells were seeded in 24-well tissue culture plates (5.0 × 10^4^ cells/well). After confirming 100% confluence, chambers with CS or NP containing 1 μg of rhBMP-2 were suspended above each well. The medium was replaced three times/week.

##### ALP staining and activity

ALP staining and activity were evaluated after 1, 7, and 14 days of co-culture. Cultured cells were fixed with 4% paraformaldehyde and overlaid with 5-bromo-4-chloro-3-indolyl-phosphate and nitro blue tetrazolium. ALP staining was quantified by gray values and ImageJ software (version 1.52q, U. S. National Institutes of Health, Bethesda, MD, USA). ALP activity was quantified with the LabAssay ALP kit (Wako Pure Chemical Industries, Ltd., Osaka, Japan) and standardized to whole-protein content measured with a bicinchoninic acid protein assay kit (Thermo Fisher Scientific, Waltham, MA, USA).

##### Quantitative real-time PCR

Cultured cells were homogenized with zirconia beads and TRIzol Reagent. Total RNA was extracted with a Direct-zol RNA kit (Zymo Research, Tustin, CA, USA) and reverse transcribed to cDNA with ReverTra Ace qPCR RT Master Mix. Gene expressions of osteogenic genes (Runx2, Osx, and Ocn) and glyceraldehyde 3-phosphate dehydrogenase (GAPDH) were quantified by real-time PCR with SYBR green master mix with a StepOnePlus Real-Time PCR System (see Supplementary Table 1 for primer sequences). Target gene expression levels were normalized to those of GAPDH, and fold changes were calculated relative to the control group by the 2^−∆∆Ct^ method.

##### Immunocytochemical analysis

The osteogenic genes Runx2 and Ocn were stained. Cultured cells were fixed with 4% PFA, blocked with PBS containing 0.2% Triton X-100 and 5% BSA, incubated overnight at 4 °C with first antibodies against Runx2 (Abcam, Cambridge, UK; ab192256, 1:1000) and Ocn (Takara Bio Inc, Shiga, Japan; 1:100) and then stained with Alexa Fluor Plus 488–conjugated goat anti-rabbit secondary antibody and Alexa Fluor 555–conjugated goat anti-rat secondary antibody (Invitrogen, Waltham, MA, US; A32731 and A21434, 1:1000) for 1 h. Cell nuclei were stained with DAPI solution and mounted with Prolong Diamond Antifade Mountant, and fluorescence images were acquired with a BZ-X700 All-in-One Fluorescence Microscope.

### Posterolateral spinal fusion model

Twenty-two 8-week-old male Sprague Dawley rats underwent posterolateral spinal fusion (see below) and were divided into a CS group (CS containing 1 μg of rhBMP-2, *n* = 11) and an NP group (NP containing 1 μg of rhBMP-2, *n* = 11). The number of animals required was calculated from the results of a preliminary experiment and our previous studies^33,38^. At postoperative week 8, rats were euthanized by anesthetic overdose, and treated spinal segments were harvested and evaluated by high-resolution micro-CT and histological analysis.

### L4–L5 posterolateral spinal fusion

A posterior midline incision was made on the skin and two paramedian incisions were made in the lumbar fascia 3 mm from the midline, exposing the L4 and L5 transverse processes^33,38^ (Supplementary Fig. 1a,b). The transverse processes were decorticated with a high-speed burr (Supplementary Fig. 1c). Blood oozed from the bone marrow (Supplementary Fig. S1d), and CS or NP was implanted on each side of the vertebrae (Supplementary Fig. 1e).

### Preparation of carrier material

CS: A CS sheet was cut into a 5 × 10 mm^2^ rectangle; 1 μg of rhBMP-2 was dissolved in 100 μL of PBS and applied to the CS immediately before implantation.

NP: A 5 × 10 mm^2^ rectangle of NP containing 1 μg of rhBMP-2 was prepared.

### High-resolution micro-CT analysis

At postoperative week 8, the explanted spine was scanned by high-resolution micro-CT (SkyScan 1272). Spinal fusion was defined as the formation of bone with cortical continuity between L4 and L5 transverse processes on either the left or right^33,38^ (Supplementary Fig. 1f.). To evaluate the bone quality of successfully fused spinal fusion masses, we analyzed BMD, as follows: A 1 × 1 ×   mm^3^ rectangular ROI passing through the center of the spinal fusion mass was placed cranial to the L4/L5 disc in the coronal plane and ventral to the posterior margin of the vertebral body in the axial plane (Fig. 4b). BMD in the ROI was measured with CTAn software (Bruker Corporation, Billerica, MA, USA).

#### Manual palpation test

Three independent examiners manually tested explanted spines, evaluated intersegmental motion and rated spines as fused or not. A failure of fusion was defined as any motion on either side between the facets or transverse processes^33,38^. Spinal segments were considered fused only if all three examiners agreed.

#### Histological analysis

Dissected and formalin-fixed spinal segments were decalcified by K-CX solution, dehydrated with an ethanol series and embedded in paraffin wax. Next, 5-μm thick coronal sections were cut at the level of the anterior one-third of the vertebral body. H&E, safranin-O, and Goldner’s Masson trichrome staining and osteocalcin immunostaining (1:200, bs-4917R) were performed according to the manufacturers’ instructions.

### Histological quantification of newly formed trabecular bone

Newly formed trabecular bone inside the fused spinal fusion masses between L4 and L5 transverse processes was quantified by H&E histological sections. A 1 × 2 mm^2^ ROI was placed cranial to L4/L5 at the spinal fusion area. Trabecular bone in the ROI was color coded and measured with ImageJ software (version 1.52q, U. S. National Institutes of Health)^40^, and the percentage of trabecular bone in the ROI was calculated.

### Statistical analysis

Two groups were compared by an unpaired Student’s *t* or Mann–Whitney *U* test; and 3 groups, by one-way analysis of variance and Bonferroni multiple comparison. Fisher’s exact test was used to compare spinal fusion rates. Data were expressed as means ± S.D. and analyzed by GraphPad Prism 8.0. A *p* value less than 0.05 was considered statistically significant.

## Supplementary Information

Supplementary Information.

## Data Availability

The datasets generated and/or analyzed during the current study are available from the corresponding authors on reasonable request.
