# Supplementary material for: A novel BMP-2–loaded hydroxyapatite/beta-tricalcium phosphate microsphere/hydrogel composite for bone regeneration
Source: Sci Rep. 2021 Aug 19;11:16924. doi: 10.1038/s41598-021-96484-4 (PMC8376985; doi:10.1038/s41598-021-96484-4)
Supplement: Supplementary file 1 — Supplementary Information. [file 41598_2021_96484_MOESM1_ESM.pdf]

Title

A novel BMP-2–loaded hydroxyapatite/beta-tricalcium phosphate microsphere/hydrogel composite for bone regeneration

Authors

Daisuke Tateiwa[1] MD, Shinichi Nakagawa[1] MD, Hiroyuki Tsukazaki[1] MD, Rintaro Okada[2] MD, PhD, Joe Kodama[3] MD, Junichi Kushioka[1] MD, PhD, Zeynep Bal[1] MSc, Yuichiro Ukon[1] MD, Hiromasa Hirai[1] MD, Takashi Kaito[1]* MD, PhD

Affiliations

[1]Department of Orthopedic Surgery, Osaka University Graduate School of Medicine, Suita, Osaka, Japan

[2]Department of Orthopedic Surgery, Mino Municipal Hospital, Mino, Osaka, Japan

[3]Department of Orthopedic Surgery, Kansai Rosai Hospital, Amagasaki, Hyogo, Japan

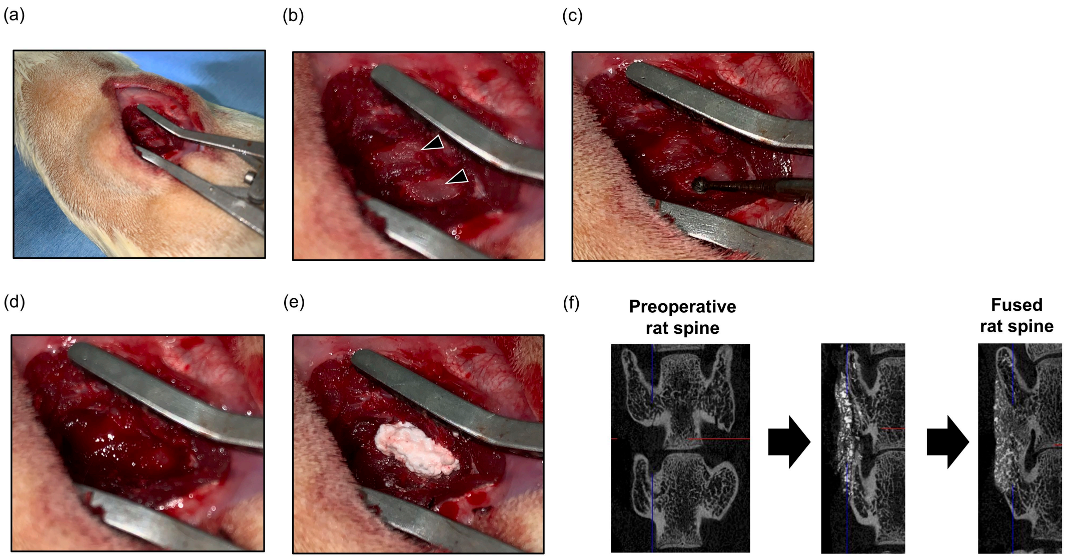

Supplementary Figure 1. Surgical procedure of L4–L5 posterolateral spinal fusion. (a, b) A posterior midline skin incision was made, followed by paramedian incisions in the lumbar fascia, exposing the L4 and L5 transverse processes (black arrows). (c) Decortication of L4 and L5 transverse processes using a high-speed burr. (d) Blood oozing from the bone marrow. (e) Implantation of NP. (f) Spinal fusion was defined as a bridging bone with cortical continuity between the L4 and L5 transverse processes.

Supplementary Table 1 Primers used in this study

Gene	Forward	Reverse
Runx2	AGGCACAAAGAAGCCATAC	AATGAGTGAGGGAAGGGT
OSX	CATCTGCCTGACTCCTTGGGAC	GCTGAAAGGTCAGCGTATGGC
OCN	TGGCGACACTTACCGAGCTT	CCATGCCCCTTGTAGTAGCTGTA
GAPDH	GGGTGTGAACCACGAGAAAT	ACTGTGGTCATGAGCCCTTC
